# Physiological Levels of Nitric Oxide Diminish Mitochondrial Superoxide. Potential Role of Mitochondrial Dinitrosyl Iron Complexes and Nitrosothiols

**DOI:** 10.3389/fphys.2017.00907

**Published:** 2017-11-07

**Authors:** Sergey I. Dikalov, Vladimir I. Mayorov, Alexander V. Panov

**Affiliations:** ^1^Department of Medicine, Vanderbilt University Medical Center, Nashville, TN, United States; ^2^Division of Basic Medical Sciences, Mercer University School of Medicine, Macon, GA, United States; ^3^Institute of Molecular Biology and Biophysics, Russian Academy of Sciences, Novosibirsk, Russia

**Keywords:** mitochondria, superoxide, nitric oxide, dinitrosyl iron complexes, nitrosothiols, electron spin resonance

## Abstract

Mitochondria are the major source of superoxide radicals and superoxide overproduction contributes to cardiovascular diseases and metabolic disorders. Endothelial dysfunction and diminished nitric oxide levels are early steps in the development of these pathological conditions. It is known that physiological production of nitric oxide reduces oxidative stress and inflammation, however, the precise mechanism of “antioxidant” effect of nitric oxide is not clear. In this work we tested the hypothesis that physiological levels of nitric oxide diminish mitochondrial superoxide production without inhibition of mitochondrial respiration. In order to test this hypothesis we analyzed effect of low physiological fluxes of nitric oxide (20 nM/min) on superoxide and hydrogen peroxide production by ESR spin probes and Amplex Red in isolated rat brain mitochondria. Indeed, low levels of nitric oxide substantially attenuated both basal and antimycin A-stimulated production of reactive oxygen species in the presence of succinate or glutamate/malate as mitochondrial substrates. Furthermore, slow releasing NO donor DPTA-NONOate (100 μM) did not change oxygen consumption in State 4 and State 3. However, the NO-donor strongly inhibited oxygen consumption in the presence of uncoupling agent CCCP, which is likely associated with inhibition of the over-reduced complex IV in uncoupled mitochondria. We have examined accumulation of dinitrosyl iron complexes and nitrosothiols in mitochondria treated with fast-releasing NO donor MAHMA NONOate (10 μM) for 30 min until complete release of NO. Following treatment with NO donor, mitochondria were frozen for direct detection of dinitrosyl iron complexes using Electron Spin Resonance (ESR) while accumulation of nitrosothiols was measured by ferrous-N-Methyl-D-glucamine dithiocarbamate complex, Fe(MGD)_2_, in lysed mitochondria. Treatment of mitochondria with NO-donor gave rise to ESR signal of dinitrosyl iron complexes while ESR spectra of Fe(MGD)_2_ supplemented mitochondrial lysates showed presence of both dinitrosyl iron complexes and nitrosothiols. We suggest that nitric oxide attenuates production of mitochondrial superoxide by post-translational modifications by nitrosylation of protein cysteine residues and formation of protein dinitrosyl iron complexes with thiol-containing ligands and, therefore, nitric oxide reduction in pathological conditions associated with endothelial dysfunction may increase mitochondrial oxidative stress.

## Introduction

Antioxidant properties of endothelial-derived nitric oxide (NO^•^) are strongly associated with inhibition of platelet activation, suppression of vascular smooth muscle proliferation, inhibition of leukocyte adherence (Somers and Harrison, 1999) and reduction of myocardial injury during ischemia(Wolfrum et al., 2003). Decreased NO^•^ production by endothelial nitric oxide synthase (eNOS) constitutes an early step in the pathogenesis of vascular disease (Harrison and Cai, 2003) and is associated with increased superoxide (O_2_^•^) generation (Spiekermann et al., 2003). However, little is known about regulation of O_2_^•^ production by NO^•^.

Nitric oxide is synthesized from L-arginine by three major isoforms of NO synthase eNOS, iNOS, nNOS, and, possibly, by mitochondrial isoform (mtNOS), but its properties still remain uncertain. NO^•^ can rapidly react with O_2_^•^ to produce highly reactive peroxynitrite. However, NO^•^ is highly lipophilic molecule, therefore, due to its high concentration in the hydrophobic environment (lipid membrane and proteins) it can exert its regulatory effects via heme nitrosylation, binding to FeS centers and S-nitrosylation (Kagan et al., 2001; Gow et al., 2002; Pieper et al., 2003). It has been demonstrated that reduced bioavailability of tetrahydrobiopterin, a critical co-factor for eNOS, and L-arginine, eNOS substrate, lead to eNOS “uncoupling” which produces O_2_^•^ rather than NO^•^ (Xia et al., 1996; Kuzkaya et al., 2003). Impaired flow-dependent endothelium-mediated vasodilatation in cardiovascular patients and metabolic conditions, at least in part, occurs due to accelerated degradation of nitric oxide and diminished nitric oxide production (Landmesser et al., 2002; Walther et al., 2015). Diminished nitric oxide levels increases intracellular Ca^2+^ due to impaired cellular cGMP pathway and reduced NO-mediated post-translational modifications leading to cellar dysregulations (Adachi et al., 2004; Thomas et al., 2006).

Mitochondrial O_2_^•^ plays an important role in the ischemia/reperfusion injury, neurodegeneration and aging (Cadenas and Davies, 2000). We have recently demonstrated an important role of mitochondrial O_2_^•^ in endothelial dysfunction and hypertension (Dikalova et al., 2010; Dikalov et al., 2014). We hypothesize that reduced nitric oxide levels may contribute to elevation in mitochondrial O_2_^•^. Indeed, endothelial dysfunction is accompanied by increased intracellular Ca^2+^ and deletion of Ca^2+^ sensitive regulatory subunit of the mitochondrial permeability transition pore Cyclophilin D reduces mitochondrial O_2_^•^, improves endothelial function and attenuates hypertension (Kroller-Schon et al., 2014; Itani et al., 2016). Complex I S-nitrosylation is cardio-protective (Burwell et al., 2006; Nadtochiy et al., 2007) and mitochondrial nitroso-proteomes reveal that endogenous NO is associated with SNO-proteins in energy and redox regulation, transport, iron homeostasis, translation, mitochondrial morphology, and apoptosis (Satohisa et al., 2014).

The effect of NO^•^ on mitochondria is still controversial. Some studies show inhibition of mitochondrial respiration by nitric oxide (Galkin and Moncada, 2007; Moncada, 2015). This effect however was observed at high nitric oxide or SNO donor levels. We suggested that normal low physiological levels of nitric oxide should not cause mitochondrial dysfunction and may potentially reduce production mitochondrial O_2_^•^ via post-translational modifications of mitochondrial proteins. We have previously measured physiological NO^•^ production in aorta as 20 nM/min (Dikalov and Fink, 2005). In this work we have investigated the effect of low physiological fluxes of NO^•^ on O_2_^•^ and H_2_O_2_ production, respiration and accumulation of mitochondrial dinitrosyl iron complexes and nitrosothiols.

## Materials and methods

### Animal use and ethics statement

All animal experiments were performed in accordance with NIH Guide for the Care and Use of Laboratory Animals and approval by the Institutional Animal Care and Use Committee at Emory University and Vanderbilt University Medical Center. All surgery was performed under anesthesia and all efforts were made to minimize animal suffering. Two- to three-month-old male Sprague-Dawley rats and male C57Bl/6J mice were used for isolation of the kidney and brain mitochondria.

### Isolation of brain and kidney mitochondria

Both brain and kidney mitochondria were isolated in medium that contained (in mM) 225 mannitol, 75 sucrose, 20 MOPS (pH 7.2), 1 EGTA, and 0.1% BSA. Kidney mitochondria (LM) were isolated by conventional differential centrifugation with a final spin at 8,600 g. Brain mitochondria were isolated from the pooled forebrains of three rats. We used the modified method of Sims (53) to isolate and purify brain mitochondria (BM) in a Percoll gradient. The modifications were as follows: brain tissue was homogenized with 15 strokes of a loose pestle in a Dounce homogenizer, and 5-ml volumes per tube of 15, 23, and 40% (vol/vol) of Percoll solutions were used to purify the brain mitochondria. After the final sedimentation of mitochondria at 8,600 g, the mitochondria were suspended in 250 mM sucrose and 10 mM MOPS (pH 7.2) (Panov et al., 2007).

### Measurements of H_2_O_2_ release by mitochondria

H_2_O_2_ was determined using Amplex red (Molecular Probes) method. In the presence of horseradish peroxidase, the following reaction occurs: Amplex red + H_2_O_2_ → resorufin + O_2_. Resorufin is a stable and highly fluorescent compound with a wavelength spectra excitation/emission of 570/585 nm. The fluorescence of resorufin was determined in 1-ml incubations in a medium (*medium A*) containing (in mM) 125 KCl, 10 MOPS, pH 7.2, 2 MgCl_2_, 2 KH_2_PO_4_, 10 NaCl, 1 EGTA, 0.7 CaCl_2_, and 0.2 mg/ml mitochondrial protein, 5 μM Amplex red, and 3 units of horseradish peroxidase, as previously described (Panov et al., 2007). We measured H_2_O_2_ production in the presence of glutamate 20 mM + malate 2 mM, or succinate 5 mM. Fluorimetric measurements were made using a fluorometer from C&L (Middletown, PA).

### Measurements of O_2_^•^ release by mitochondria

The cyclic hydroxyl-amine PPH (Enzo Life Sciences, Inc., Farmingdale, NY) was used for measurements of O_2_^•^ release by mitochondria (Panov et al., 2005). PPH reacts with O_2_^•^ producing stable PP-nitroxide detected with ESR spectroscopy (Dikalov et al., 2011). Briefly, 10 mM PPH was dissolved in deoxygenated media with 50 μm deferoxamine. Mitochondria preparations and PPH stock solutions were kept on ice (50 μg of protein mixed with 1 mm PPH and mitochondrial substrates in 100 μl of Medium. Detection of radical was confirmed by inhibition of the ESR signal with 50 units/ml of SOD. Accumulation of PP-nitroxide was measured using a Bruker EMX ESR spectrometer. Superoxide production was detected by following the low-field peak of the nitroxide ESR spectra using time scans with the following ESR settings: microwave frequency 9.78 GHz, modulation amplitude 2 G, microwave power 10 dB, conversion time 1.3 s, and time constant 5.2 s.

### Statistics

Data are presented as a mean ± S.E. for four or five separate measurements of a parameter. For comparison of two groups, a two-tailed *t*-test was employed using Excel software. Statistical significance was assumed when *p* < 0.05.

## Results

### Effect of low nitric oxide on O_2_^•^ release by rat brain mitochondria

**Figure 1:**
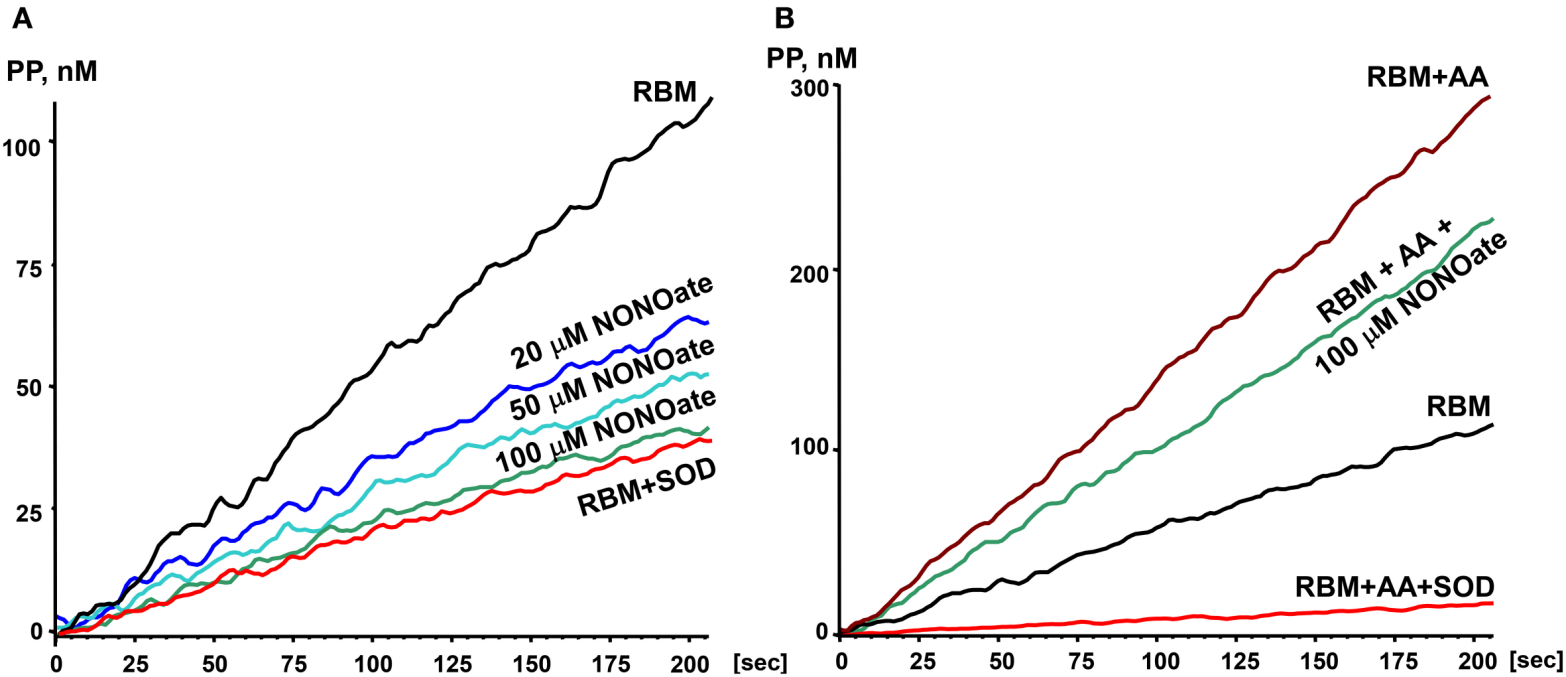
Effect of nitric oxide on O_2_^•^ release by rat brain mitochondria. Rat brain mitochondria (RBM) were placed in respiration media with Glutamate/Malate as a substrate and acutely treated with slow releasing NO-donor DPTA Nonoate **(A)**. To test the potential effect of NO on complex III-mediated O_2_^•^ production mitochondria were supplemented with complex III blocker Antimycin A (1 μM) **(B)**. Release of mitochondrial O_2_^•^ was measured by spin probe PPH (1 mM) and accumulation of PP-nitroxide followed by ESR spectrometer as described in Material and Methods. Addition of Cu,Zn-superoxide dismutase (SOD, 10 Units/ml) confirms specific detection of extramitochondrial O_2_^•^. Figure shows typical ESR data of four independent experiments.

In order to study the effects of NO on O_2_^•^ production by mitochondria we used the slow releasing NO-donor DPTA-NONOate (half-life time is 5 h) (Keefer et al., 1996). We have determined that 100 μM DPTA-NONOate generates NO at 20 nM/min which corresponds to normal endothelial NO production (Dikalov and Fink, 2005). Our experiments with rat brain mitochondria demonstrated strong inhibition of O_2_^•^ production by 20 μM NONOate (Figure 1A). Inhibition was concentration dependent and reached maximum at 100 μM NONOate (Figure 1A). It was found that NONOate only partially inhibited mitochondrial O_2_^•^ production stimulated by Antimycin A (Figure 1B). Of note, the amount of inhibited O_2_^•^ in the presence of Antimycin A was equivalent to the amount of O_2_^•^ blocked in the absence of Antimycin A (Figures 1A,B). It is important to note that NO can potentially react with O_2_^•^ producing highly reactive peroxynitrite. Meanwhile, PPH spin probe detects both O_2_^•^ and peroxynitrite, therefore, potential peroxynitrite formation should not diminish the ESR signal (Dikalov et al., 1998). Furthermore, NO-mediated inhibition of O_2_^•^ production was not affected by complex III inhibitor Antimycin A despite 3-fold increase of O_2_^•^ production in the presence of Antimycin A suggesting site specific regulatory effect of NO. These data suggest that NO inhibits superoxide production by complex I but does not affect superoxide release by complex III stimulated by addition of Antimycin A.

### Effect of nitric oxide on O_2_^•^ release by rat brain mitochondria

**Figure 2:**
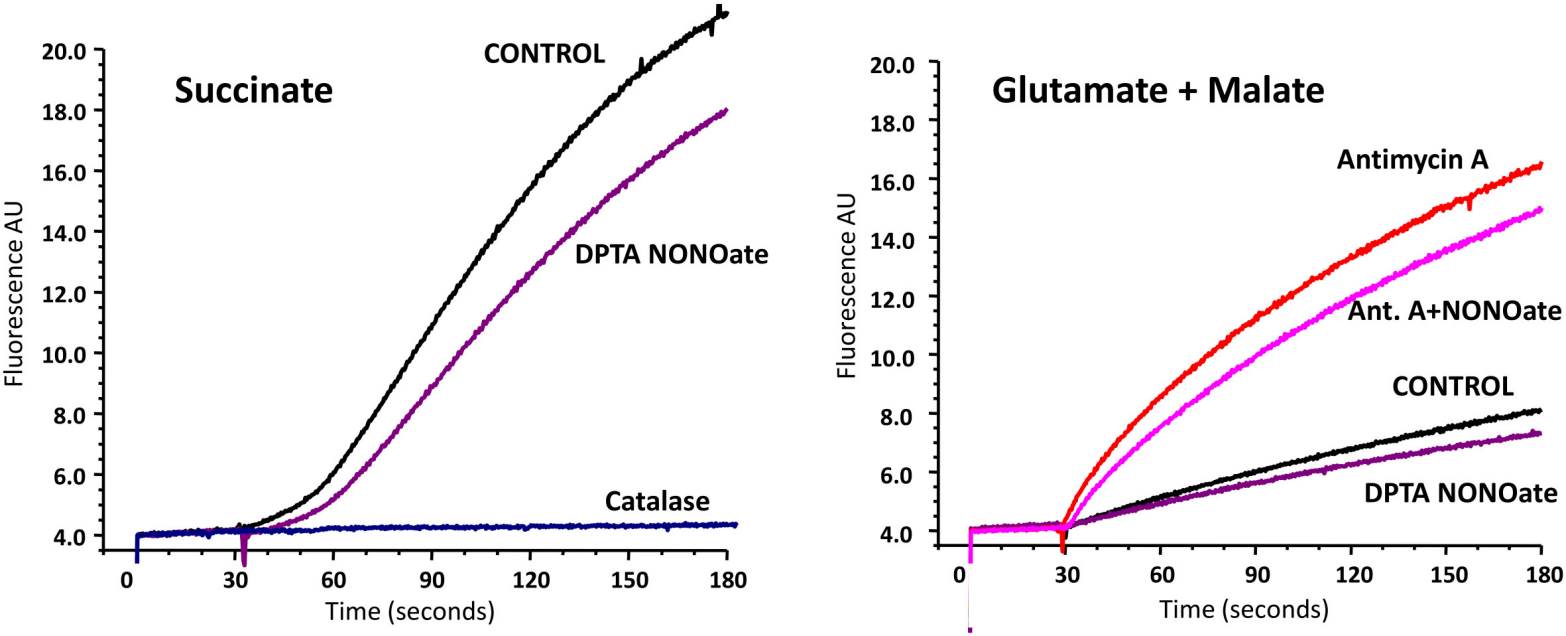
Effect of nitric oxide on H_2_O_2_ release by rat brain mitochondria measured with Amplex Red. Rat brain mitochondria (RBM) were supplemented with slow releasing NO-donor DPTA NONOate (100 μM). To test the potential effect of NO on complex III-mediated H_2_O_2_ production mitochondria were treated with complex III blocker Antimycin A (1 μM). Release of mitochondrial H_2_O_2_ was measured by Amplex Red assay as described in Material and Methods. Addition of catalase (20 μg/ml) confirms specific detection of mitochondrial H_2_O_2_. Figure shows typical data of four independent experiments.

It is known that several sites on complex I, II, and III may be involved in mitochondrial O_2_^•^ production. Most of them, however, release O_2_^•^ in the matrix, where O_2_^•^ is quickly dismutate by Mn-SOD to H_2_O_2_. Therefore, the analysis of H_2_O_2_ reflects total mitochondrial O_2_^•^ production, while extarmitochondrial O_2_^•^ detection may not take into account O_2_^•^ produced in the matrix (Figure 2). In order to test the effect of NO^•^ on total mitochondrial O_2_^•^ we measured H_2_O_2_ production by brain mitochondria in the absence and presence of NO-donor (Figure 2). It was found that NO^•^ partially inhibited H_2_O_2_ production both with succinate and glutamate/malate as mitochondrial substrates. It is interesting that inhibition of H_2_O_2_ production with succinate was similar to the effect of rotenone, which blocks O_2_^•^ production on complex I due to reverse electron flow. Inhibition of H_2_O_2_ production with glutamate/malate resembled the one observed with O_2_^•^ measurements by ESR (Figure 1). The amount of inhibited O_2_^•^ in the presence of Antimycin A was similar to the amount blocked in the absence of Antimycin A (Figure 2, Glutamate+Malate). One of the main sites of O_2_^•^ production with glutamate/malate is associated with complex I, which can generate O_2_^•^ both to the matrix and intermembrane space. It is possible that NO may inhibit O_2_^•^ production at the site facing the intermembrane space, but does not affect O_2_^•^ production in the matrix.

### Effect of slow releasing NO-donor on mitochondrial respiration

**Figure 3:**
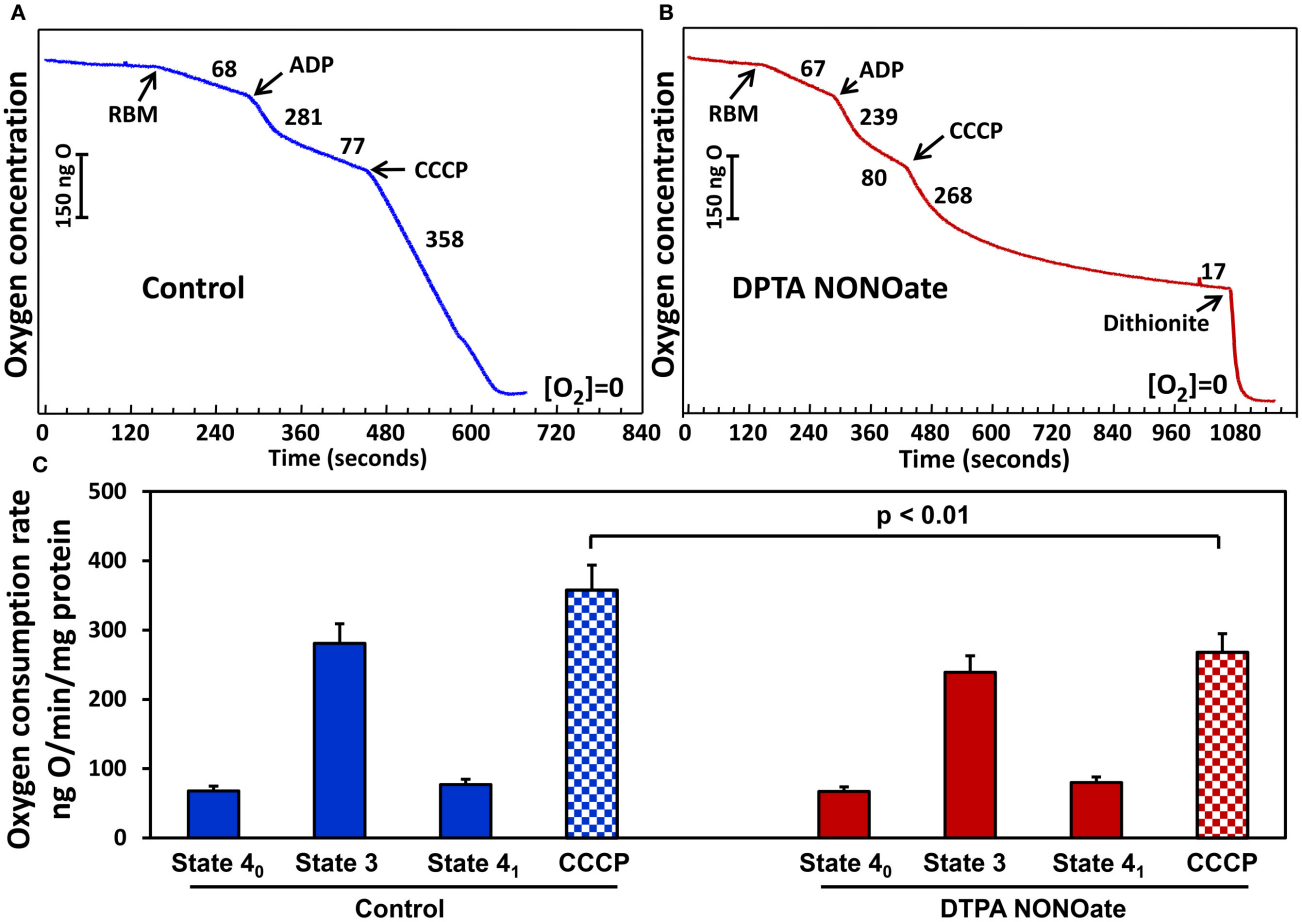
Effect of nitric oxide on respiration with glutamate/malate by rat brain mitochondria (RBM) **(A)**. Mitochondria (0.5 mg/ml) were supplemented with slow releasing NO-donor DPTA NONOate (100 μM) **(B)**. **(C)** Oxygen consumption rate is expressed as ng O_2_/min/mg protein. Clark oxygen electrode was calibrated with air saturated water at 25°C. Instrumental zero oxygen level ([O_2_] = 0) was confirmed at the end of every experiment by addition of sodium dithionite as shown in **(B)**. State 4_0_, State 3 and State 4_1_ were defined as previously described in Panov (2014). Results represent mean ± SEM (*n* = 4). ^*^P < 0.01 vs. NONOate/CCCP.

On the next step we tested if slow release DPTA-NONOate did inhibit mitochondrial respiration. Rat brain and kidney mitochondria were isolated as we have described previously (Panov et al., 2004). We have compared respiration of mitochondria with glutamate-malate in the absence or presence of DPTA-NONOate (Figure 3). It was found that 100 μM DPTA-NONOate did not significantly change oxygen consumption in State 4 and State 3. However, the NO-donor strongly inhibited oxygen consumption in the presence of uncoupling agent CCCP, which is likely associated with inhibition of the reduced complex IV. This experiment argues that NO-donor DPTA-NONOate did not affect mitochondrial respiration in State 4, and inhibition of the superoxide release was due to specific effect on superoxide production rather than inhibition of mitochondrial respiration. NO, though, rapidly inhibited the uncoupled mitochondrial respiration, when complex IV was more reduced than in State 4.

### Accumulation of dinitrosyl iron complexes and nitrosothiols in NO-donor treated mitochondria

**Figure 4:**
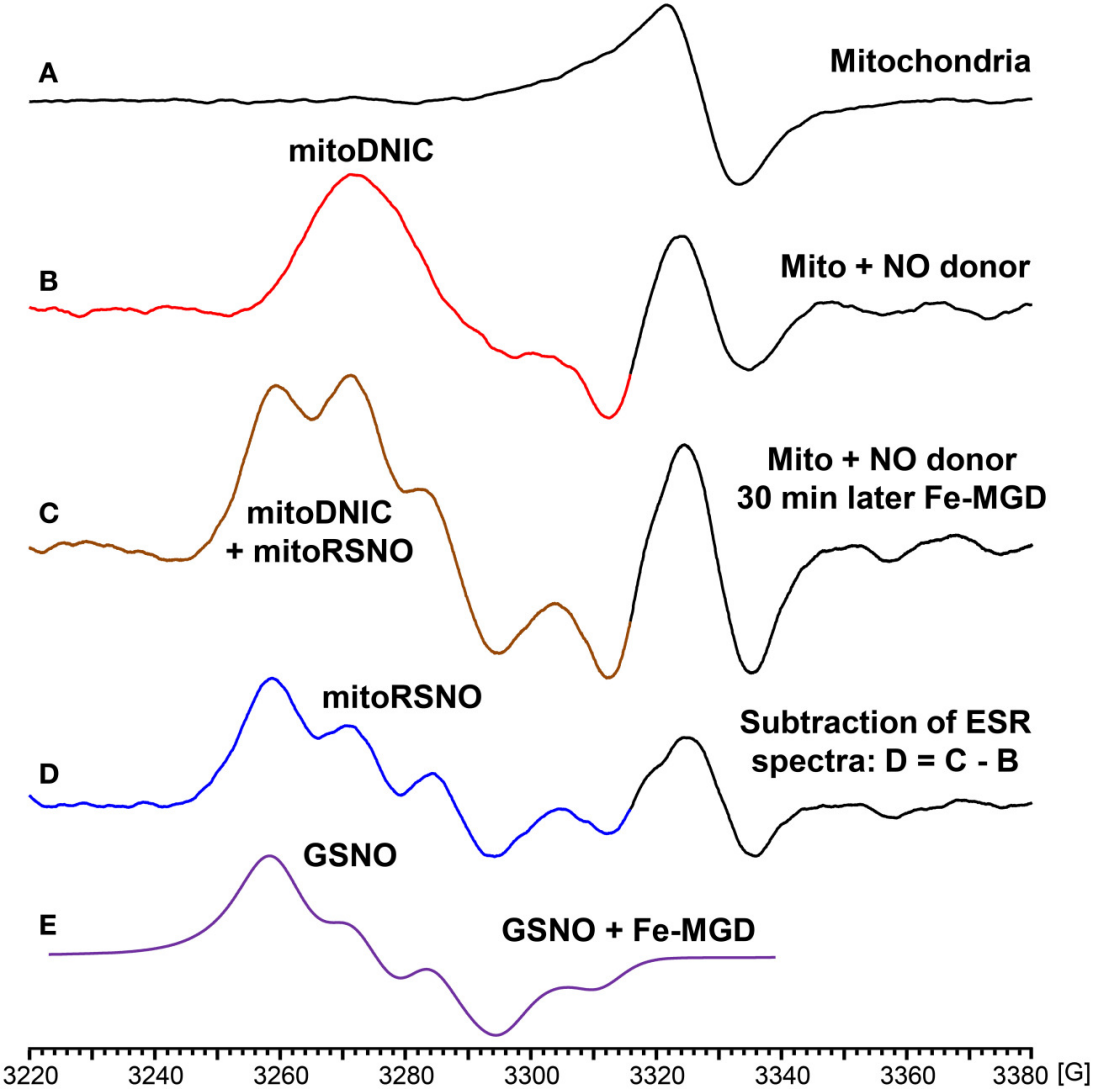
Formation of dinitrosyl iron complexes and nitrosothiols in nitric oxide-treated mitochondria. Mitochondria were isolated from mouse kidney (10 mg/ml) and incubated in respiration media at 25°C for 30 with vehicle or NO-donor MAHMA Nonoate (10 μM). Then mitochondria were snap-frozen in liquid nitrogen for Electron Spin Resonance (ESR) studies. **(A)** Untreated mitochondria; **(B)** NO-donor treated mitochondria; **(C)** Lysed NO-donor treated mitochondria with 0.1 mM Fe(MGD)_2_ complex; **(D)** Subtraction of dinitrosyl iron complex ESR spectrum **(B)** from spectrum **(C)** revealed the presence of mitochondrial nitrosothiols (mitoRSNO); **(E)** Reference ESR spectrum of 2 μM GSNO plus 0.1 mM Fe(MGD)_2_. The average integral amount of mitochondrial dinitrosyl iron complexes (mitoDNIC) and nitrosothiols ESR signal is 2 μM (standard error <15%). Figure shows typical ESR spectra of four independent experiments.

In additional experiments we have studied NO-mediated post-translational modifications in mitochondria. For this aim we have treated isolated mouse kidney mitochondria with fast releasing NO donor MAHMA-NONOate (half-life time is 3 min) (Keefer et al., 1996). Mitochondria were incubated with 10 μM MAHMA-NONOate for 30 min until complete release of NO which was confirmed in the separate experiments in respiration media without mitochondria using NO spin trap Fe(MGD)_2_ (Komarov et al., 2000). Following incubation with MAHMA-NONOate or vehicle mitochondria (10 mg protein/ml) were placed in insulin syringes and snap frozen in the liquid nitrogen for ESR analysis. Untreated mitochondria show characteristic heme spectrum (Figure 4A), however, NO-donor treated mitochondria showed a robust accumulation of dinitrosyl iron complexes with thiol-containing ligands (Figure 4B) detected by specific ESR spectrum of dinitrosyl iron complexes as has been previously reported by Dr. Vanin (Vanin, 2016). In order to test the accumulation of nitrosothiols we have lysed mitochondria by freeze-thaw cycles and added 0.1 mM Fe(MGD)_2_ which rapidly converts SNO to NO-Fe(MGD)_2_ complex detectable by ESR (Komarov et al., 2000; Vanin et al., 2004). Indeed, supplementation of Fe(MGD)_2_ to NO-treated mitochondria substantially changed ESR spectra by adding the specific triplet ESR signal of NO-Fe(MGD)_2_ complex (Figure 4C). Additional analysis of ESR spectra showed the presence of both dinitrosyl iron complexes and nitrosothiols in NO-donor treated mitochondria (Figures 4B,D). To quantify the accumulation of dinitrosyl iron complexes and nitrosothiols in NO-donor treated mitochondria we have used reference calibration sample containing 2 μM GSNO plus 0.1 mM Fe(MGD)_2_ (Figure 4E). Analysis of double integral intensities of ESR spectra showed that the accumulation of dinitrosyl iron complexes and nitrosothiols in MAHMA-NONOate treated mitochondria is close to 2 μM.

## Discussion

This study provides the first evidence that physiological levels of nitric oxide reduce mitochondrial superoxide production without significant inhibition of mitochondrial respiration. We have employed two independent assays for measurements of mitochondrial reactive oxygen species. Analysis of superoxide release was done by mitochondria-impermeable spin probe PPH while release of H_2_O_2_ was measured by Amplex Red assay (Panov et al., 2005). It was found that low physiological levels of nitric oxide reduced both O_2_^•^ and H_2_O_2_ production. Our data show that treatment of mitochondria with NO-donor lead to significant accumulation of dinitrosyl iron complexes and nitrosothiols suggesting the role of NO-mediated post-translational modifications in down-regulation of mitochondrial O_2_^•^ production.

Physiological production of nitric oxide is mainly mediated by endothelial nitric oxide synthase (eNOS) in the vasculature and kidney while neural nitric oxide synthase (nNOS) is important in the brain, heart and skeletal muscle (Silberman et al., 2010; Gonzalez et al., 2015). It is interesting that these tissues are highly metabolically active and it appears to develop adaptation to avoid the interference of low fluxes of nitric oxide with mitochondrial respiration. Furthermore, nitric oxide in these tissues modulates mitogenesis and reduces intracellular Ca^2+^ which attenuates mPTP opening and improves mitochondrial function (Hassid et al., 1994; Khan and Hare, 2003; Lira et al., 2010). Our data show that nitric oxide gas in mitochondria is converted into nitrosyl, NO+, which does not affect respiration and mediates post-translational modifications of cysteine residues into dinitrosyl iron complexes and nitrosothiols. Our ESR study of nitrosylation of mitochondrial targets is consistent with previous ESR analysis of cellular dinitrosyl iron complexes known as DNIC (Kleschyov et al., 2007). There are low molecular DNIC complexes such as low molecular weight DNIC–cysteine and protein DNIC. Our preliminary data showed accumulation of DNIC and nitrosothiols both in the mitochondrial membrane fraction and mitochondrial matrix (supernatant) suggesting nitrosylation of low molecular and protein targets. It has been previously shown that S-nitrosylation of NADPH oxidase reduces O_2_^•^ production (Selemidis et al., 2007) and we suggest that similar post-translational modifications may contribute to down-regulation of mitochondrial O_2_^•^. The precise molecular mechanisms of “anti-oxidant” effect of NO in mitochondria are not clear and it may include not only inhibition of O_2_^•^ production but also upregulation of mitochondrial antioxidants such as superoxide dismutase and redox buffering of peroxides.

The discrepancy that exists in the literature about contribution of respiratory chain complexes I and III into mitochondrial ROS production to a large extent can be explained by the fact that mitochondria from different organs have different rates of State 4 and State 3 respiration and different regulatory mechanisms, as well as, different antioxidant activities (Herrero and Barja, 1997). Cardiac and skeletal muscles have antioxidant concentrations more than one order of magnitude lower than those of other highly aerobic tissues like liver or kidney (Herrero and Barja, 1997). Another important issue in studies of ROS generation is the location of the site of O_2_^•^ generation. The sites that are located close to the inner surface of the mitochondrial membrane will more likely release O_2_^•^ into the matrix, whereas the sites located close to the intermembrane space, will more likely release O_2_^•^ into the cytosolic side. Therefore, extramitochondrial probes register only O_2_^•^ that was formed close to the intermembrane space. Meanwhile, H_2_O_2_ is a neutral molecule and will easily leave mitochondria regardless of mitochondrial energization. Indeed, we have found that in brain mitochondria measurements of total ROS generation can be best followed by H_2_O_2_ production. Thus, in order to study NO-mediated modulation of mitochondrial reactive oxygen species we have investigated both O_2_^•^ and H_2_O_2_ production by mitochondria. Our data show that the amount of inhibited O_2_^•^ in the absence of Antimycin A was similar to the amount blocked in the presence of Antimycin A which is consistent with modulation of mitochondrial complex I by nitrosylation leading to reduced O_2_^•^ generation at this site.

The previous report show that high levels of DNIC (100 μM) induces apoptosis (Kleschyov et al., 2006). Indeed, inflammation is accompanied with robust activation of inducible nitric oxide synthase (iNOS) leading to NO overproduction, nitrosative stress and metabolic dysfunction (Kaneki et al., 2007). It is important to note that accumulation of mitochondrial DNIC and nitrosothiols in our experiments with physiological fluxes of NO^•^ did not exceed 2 μM and DNIC at concentrations <10 μM did not induce apoptosis (Kleschyov et al., 2006). One should take into account substantial differences between NO^•^ and NO^+^ (DNIC and nitrosothiols) in reactivity and life-time. NO^•^ is much more reactive and has a shorter life-time compared to NO^+^. Therefore, micromolar concentrations of bolus NO^•^ have very distinct effect compared to NO^+^. It has been shown that NO^•^ (bolus 1 μM) significantly increases production of O_2_^•^ and H_2_O_2_ by mitochondria (Riobo et al., 2001). It has been found that NO^•^ causes nitrosylation of complex IV, modifications of iron-sulfur clusters of complex I, S-nitrosation of cysteine residues, glutathionylation of cysteine residues, N-nitrosation of secondary amines, nitration of tyrosine residues (Brown and Borutaite, 2001, 2004). This nitrosative stress is mediated by NO^•^-derived reactive nitrogen species such as, ^•^ NO_2_, N_2_O_3_, and ONOO^−^. It has been suggested that ONOO^−^-mediated complex I nitration mimics rotenone action (Riobo et al., 2001). However, the exact mechanism of the NO^•^-mediated increase in O_2_^•^ production by mitochondria is not clear. Our own preliminary data have shown that the inhibitory effect of NO^•^ on complex IV strongly depends on the metabolic state of mitochondria: NO^•^ inhibits respiration when mitochondria have low membrane potential and complex IV is present mainly in reduced form. It is important to note that high doses of NO^•^ cause nitrosative stress, while smaller amounts of NO^•^ may have an antioxidant action. Indeed, our data showed that low physiological fluxes of NO^•^ (20 nM/min) reduced O_2_^•^ release by mitochondria respiring in the resting metabolic state (State 4), and significantly inhibited H_2_O_2_ production. This effect was likely associated with NO^•^, but not with ONOO^−^. Thus, the effect of NO^•^ on mitochondria may depend not only on the amount of NO^•^ but also on the functional state and antioxidant status of mitochondria. Of note, the effect of NO^•^ on mitochondria can depend on the type of cells and species because of organ-specific and species-specific variability of mitochondrial function (Panov et al., 2007). However, factors, which determine the balance of antioxidant/pro-oxidant action of NO^•^, are not well-defined.

It is interesting that targeting mitochondrial O_2_^•^ improves endothelial function and reduces hypertension (Dikalova et al., 2010, 2017). It is conceivable that decreased NO level in cardiovascular conditions (Landmesser and Harrison, 2001) may contribute to mitochondrial dysfunction and strategies to improve NO-production by eNOS such as tetrahydrobiopterin supplementation can also improve mitochondrial function. On the other hand, a number of pathological conditions dealing with hepatotoxicity and neurodegeneration are associated with NO^•^ overproduction by iNOS as well as with the increased oxidative stress (Venkatraman et al., 2004) and, therefore, specific inhibition of iNOS can be beneficial (Ljubisavljevic and Stojanovic, 2015). It is possible that effect of nitric oxide on mitochondrial functions has a bell shaped curve and, therefore, require an optimal NO level to balance the oxidative stress and metabolic activity.

## Author contributions

All authors listed have made a substantial, direct and intellectual contribution to the work, and approved it for publication.

### Conflict of interest statement

The authors declare that the research was conducted in the absence of any commercial or financial relationships that could be construed as a potential conflict of interest.
